# Revealing the Concealed: Alternatives to Random Dots for Stereograms

**DOI:** 10.3390/vision7040078

**Published:** 2023-12-17

**Authors:** Nicholas J. Wade

**Affiliations:** Psychology, Nethergate, University of Dundee, Dundee DD1 4HN, UK; n.j.wade@dundee.ac.uk

**Keywords:** random-dot stereograms, Julesz, carrier patterns, graphics, photography, photographics

## Abstract

Investigations of stereoscopic depth perception were transformed via the use of computer-generated random-dot stereograms in the 1960s. They realized Wheatstone’s wish of demonstrating binocular depth without monocular object recognition, and they have been the dominant stimulus for studying stereopsis since then. Alternative carrier patterns to random dots, based on graphics, photographs, and their combinations, are presented as anaglyphs and for free fusion. A wider range of concealed patterns can be revealed with these alternatives, and presenting them as anaglyphs can yield patterns that have visual appeal independent of the depth they conceal.

## 1. Introduction

Charles Wheatstone invented mirror and prism stereoscopes in 1832 but did not describe his experiments with them until 1838 [1,2,3,4,5]. He, like many visual scientists who followed, sought stimulus pairs (stereograms) that yield the perception of depth but are devoid of monocular allusions to objects. The stereoscopic figures he displayed were almost all simple line drawings: “For the purposes of illustration I have employed only outline figures; for had either shading or colouring been introduced it might be supposed that the effect was wholly or in part due to these circumstances, whereas by leaving them out of consideration no room is left to doubt that the entire effect of relief is owing to the simultaneous perception of the two monocular projections, one on each retina” [1] (p. 376). That is, he avoided the usual painterly techniques for conveying the impression of depth or distance so that only disparity was operating. Put another way, he sought to reveal to two eyes what was concealed from one. He could not exclude all cues to depth in the monocular views, but he could reduce them. Thus, Wheatstone could not fulfil his desire, but it was achieved over a century later by Belá Julesz [6,7,8] with the random-dot stereogram (RDS). The binocular combination of the two monocular stimuli revealed a region in depth that could not be seen by either eye alone. RDSs enabled stereoscopic depth perception to be investigated independently of monocular object recognition. In the period between Wheatstone and Julesz, several attempts were made to find stimuli that would satisfy Wheatstone’s wish.

Julesz was not the first to make stereograms using simple elements like dots. As early as 1870, Cajal used hand-drawn random dots to conceal patterns monocularly and reveal them stereoscopically [9]. The separated patterns on glass and board were photographed with a binocular camera and the two half-images were viewed in a stereoscope. A similar technique was probably devised by Mobbs in 1919 for making two random dot patterns in which the letter L was visible in depth when combined binocularly by over- or under-convergence. Dots of different sizes were drawn on one surface and then reproduced on a transparent surface placed over it, adding dots in the shape of the letter. The two surfaces were then photographed separately and mounted side-by-side [10]. Paired patterns of dots were produced by Kompaneysky [11] and by Aschenbrenner [12], which carried a face or a word, respectively, when combined in a stereoscope. Illustrations of both stereograms can be found in the works by Blundell [13] and Howard and Rogers [14]. Aschenbrenner was stimulated by his work on revealing camouflaged objects in aerial stereoscopic photographs, and he devised a novel means of simulating this using paper discards from a hole punch [15]. Revealing otherwise hidden objects in aerial stereoscopic photographs was also the stimulus that led Julesz to devise RDSs, but he was able to harness the power of computers in creating them. What distinguished his experiments was not only the use of computer-generated patterns but also an appreciation of their significance in furthering the understanding of stereoscopic vision [4,16].

**Figure 1:**
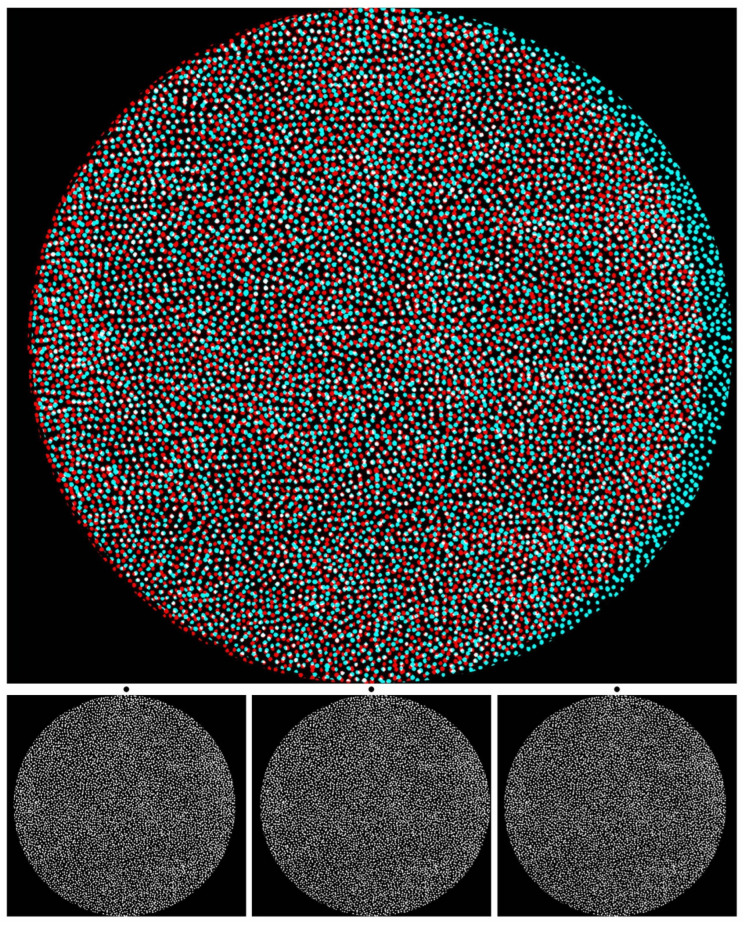
*Discord* by Nicholas Wade. (**Upper**): An anaglyph in which a central circle appears slanted in depth with the left side further and the right side nearer; the outer disc is slanted in the opposite direction. This is seen when viewed through red/cyan glasses with the red filter in front of the left (L) eye and the cyan filter in front of the right (R) eye. Reversing the filter/eye arrangement reverses the stereoscopic depths. (**Lower**): Universal Freeview arrangement of the monocular images in the sequence L–R–L. There are two ways to fuse the two stereo sets of images so as to see depth: to cross-fuse the right pair or to uncross-fuse the left pair via alignment of the dots above the appropriate monocular images. Whichever mode of viewing is adopted, the enclosed disc appears slanted in the opposite direction to the surrounding one.

Figure 1, like all the figures in this article, consists of an anaglyph above the component monocular members of the stereogram. Anaglyphs require red/cyan glasses to see the depth within them. They are presented in colours, such that one component is passed by one colour filter and blocked by the other. The first stereoscopes, invented by Wheatstone, were based on mirrors, lenses, and prisms [1,2]. The use of colours for separating the eyes to see depth was realised by Rollmann [17] in 1853 and later developed by D’Almeida [18] and by Ducos du Hauron [19], who devised a method of over-printing red and blue or green designs in 1891. Thereafter, anaglyphs became increasingly popular as a means for printing stereograms. Stereoscopes are not essential for viewing stereograms because the monocular images can be combined via over- or under-convergence. The latter is also referred to as parallel viewing. These can be achieved with the left (L) and right (R) eye components that are displayed beneath the anaglyphs in the figures that follow. The monocular images are shown in the sequence L–R–L, which is referred to as Universal Freeview. Descriptions of the depth seen in the figures will be principally in terms of the anaglyphs, but equivalent effects can be observed with appropriate crossed or uncrossed fusions of the Universal Freeview monocular stimuli. Since the monocular components are much smaller than the anaglyphs above them, it might be necessary to enlarge the images in order to extract the details for free fusion.

The hand-drawn dots in Figure 1 appear to be flat when viewed by either eye alone but are seen in depth when the upper anaglyph is viewed with a red filter in front of one eye and a cyan filter before the other. The conventional arrangement of filters and eyes for stereo photographs is red/L and cyan/R. Another way of seeing depth is by combining pairs of the lower figures which are the L–R–L eye components of the stereogram. Figure 1 is an example of an alternative to random-dot stereograms because the elements (dots) do not conform to the regular array of computer-generated RDSs. Those devised by Julesz [6] are pairs of matrices of squares in which the contents of each cell are randomly assigned as black or white. Displacing a region in one display and combining it with an untransformed image in a stereoscope results in that region appearing in depth. A variation on that theme is shown in Figure 1. Binocular viewing transforms the appearance: with a red/L and cyan/R combination, a central circle is seen slanted in depth, with its left side receding and the right side approaching, while the outer annulus appears to be slanted in the opposite direction. Reversing the filter/eye arrangement reverses the apparent depths. What was smooth and symmetrical with one eye is seen as a circle slanted in depth within the surroundings having the opposite depth. The stereoscopic depth might take some seconds to be seen, and with longer viewing, the apparently more distant side looks larger than the apparently nearer. The depth asymmetry can be induced over the whole surface (as with the slanted annulus) or in an enclosed part of it (as with the central circle). The background design in which the stereoscopic depth is embedded is called the carrier pattern [4,16]. By breaking away from the restrictions of a grid array, stereoscopic depth can be displayed more easily in the background as well as the embedded feature, and this applies to the outer annulus in Figure 1.

Presenting regular and repetitive dot patterns that enable the fusion of neighbouring pairs provides the basis of the wallpaper illusion. This binocular depth effect was initially described by Blagden [20] in the vertically fluted marble of a chimneypiece. With under-convergence so that adjacent elements were fused, the fluting appeared to be further away and magnified relative to fixating on the same elements. However, its significance was not appreciated until after the invention of the stereoscope, when Brewster [21] rediscovered the illusion when observing a repetitive pattern of flowers printed on wallpaper. It was from such patterns that were frequently printed on wallpaper that the phenomenon derived its name. When equivalent but laterally separated patterns are combined binocularly, they seem suspended in the plane of convergence. If slight variations in the locations of the repetitions along rows are introduced, then more complex depth planes are visible, and aspects of disparity processing become involved [14]. This is the principle employed in autostereograms [22,23]. Wallpaper illusions and autostereograms can be seen without the aid of any viewing device. They involve dissociating convergence from accommodation by converging the eyes to combine neighbouring elements or viewing them with parallel visual axes. This is the technique required for Universal Freeview images, and the stereograms in this article are presented in this way, beneath the anaglyphs.

A wider range of graphic designs than computer-generated random dots can act as carrier patterns for stereoscopic depth. Most graphic designs are two-dimensional. They are produced to display the interactions of contours and colours. Stereoscopic designs do not need to comply with the requirements of stereoscopic photography where objects in space do have defined distances from the camera capturing them. Variations in structure between two designs can introduce depth in ways that are difficult, if not impossible, to achieve with photographs of objects. The starting points in most of the figures that follow were graphic designs or photographs, which were then manipulated in a dark room or with computer graphics. Often the stereoscopic effect is not initially visible, and so some patience might be required for the depth to emerge. Unlike conventional stereoscopic photographs, reversing the filters or the images fused will reverse the depths seen. It will probably be the case that one eye/filter arrangement will produce more compelling depth than the other, which is a combination of eye dominance and differences between the filters [4]. In a like manner, the crossed or un-crossed fusion of the Universal Freeview images is influenced by differences between the ocular stabilities of the two eyes.

Julesz did not restrict the stereograms he made to matrices of black or white squares but produced a wider range of computer-generated carrier patterns [7]. The stereograms in the richly illustrated *Foundations of Cyclopean Perception* were presented as anaglyphs as well as stereopairs for conventional prism stereoscopes. Anaglyphs do have an advantage over paired stereograms insofar as the combined monocular views are superimposed, and that can result in designs that have visual appeal beyond the stereoscopic depth they conceal. Among the other visual scientists who were not content with simplifying visual experience was Jacques Ninio [24,25]. He has produced computer-generated patterns of stunning symmetry, which are illustrated in his books and articles. One of his books is entitled *Stéréomagie,* in which he also adapted his skills to create stereoscopic pairs as well as autostereograms. A distinction between RDSs, autostereograms, and those shown here is that the starting point for the carrier patterns is analogue rather than digital. That is, hand-drawn designs, paintings, or photographs of naturally occurring textures provide backgrounds that themselves can be modulated in depth (like the slanted outer annulus in Figure 1), or shapes within them can appear in stereoscopic depth (like the inner circle in Figure 1). The stereograms presented are based on three general categories of carrier patterns, those based on graphics, photographs, and their combinations (referred to as photographics), and examples are shown in that sequence.

## 2. Graphics

**Figure 2:**
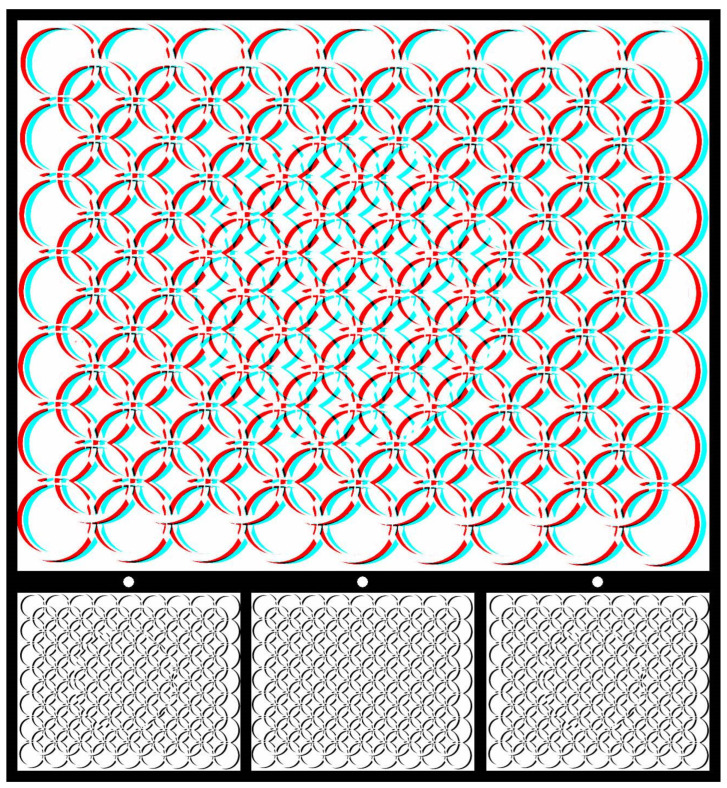
*Circularities* by Nicholas Wade.

Using graphic designs (drawings, prints, or paintings) to carry the disparities can introduce more visual appeal than the matrices of small black-and-white squares in RDSs. An example is shown in Figure 2. The pattern of interwoven circles was drawn by hand, photographed on lith film, manipulated in a darkroom, and then digitized. The large central circle was displaced in one image and combined with an untransformed pattern to produce the stereogram using StereoPhoto Maker software [26]. For the anaglyph, viewing with the red/L and cyan/R arrangement results in a central circle appearing nearer than the background, with the reverse occurring for cyan/L and red/R. Lateral head movements lead to apparent movements of the nearer circle in the same direction as the head and of the more distant one in the opposite direction. The apparently nearer circle also appears smaller than when seen further away.

**Figure 3:**
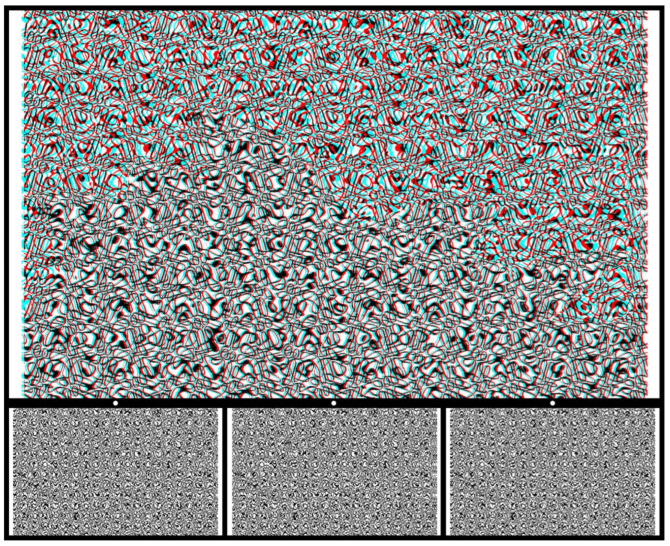
*Opera House I* by Nicholas Wade.

More complex patterns than circles and squares can be enlisted as the shapes seen in depth. The carrier pattern in Figure 3 is more complex, too, although it started out as a very simple pattern. A regular matrix of typed zeros was distorted and then superimposed upon itself to provide an irregular array of contours. A familiar structure was embedded and displaced in one pattern before combining it with the untransformed pattern. When the anaglyph is viewed through red/cyan filters, a silhouette of the sails of the Sydney Opera House will slowly emerge in depth. 

**Figure 4:**
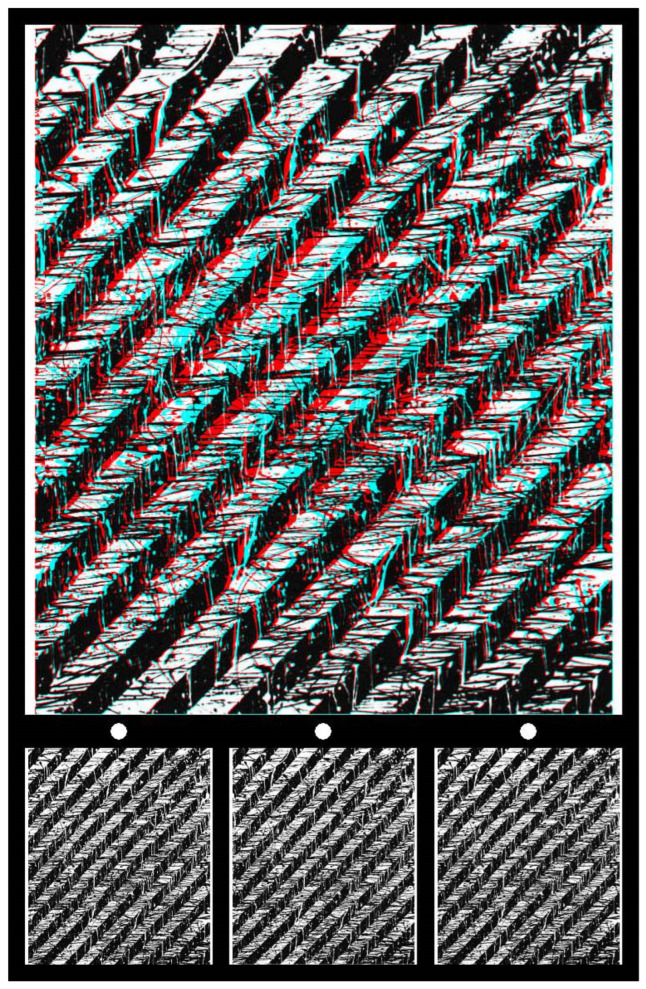
*Askew* by Nicholas Wade.

Abstract paintings can also provide a basis for creating carrier patterns. They need to fulfil the general requirements of having dense contours and being devoid of dominant pictorial features, like that in Figure 4. The painting consists of alternating black and white diagonal lines, each with streaks of their complementary colours dribbling through them, either horizontally (black) or vertically (white). Viewing the anaglyph with red/L and cyan/R, the whole surface seems to become distorted in depth, with the centre apparently more distant than the upper and lower parts. It appears to be curved towards the eyes with a reversal of the filters. What is probably not initially seen is the circle in depth at the centre of the stereogram. Once it has been seen, it will emerge quickly with subsequent viewing. As is the case for Figure 1, stereoscopic depth is modulated over the pictorial surface as well as for the circular inclusion within it. The surface of the painting appears curved in depth when viewed through red/cyan filters. With red/L and cyan/R, it appears bowed away from the viewer and the central circle looks more distant still. These depths reverse with the reversal of the eye/filter arrangement.

**Figure 5:**
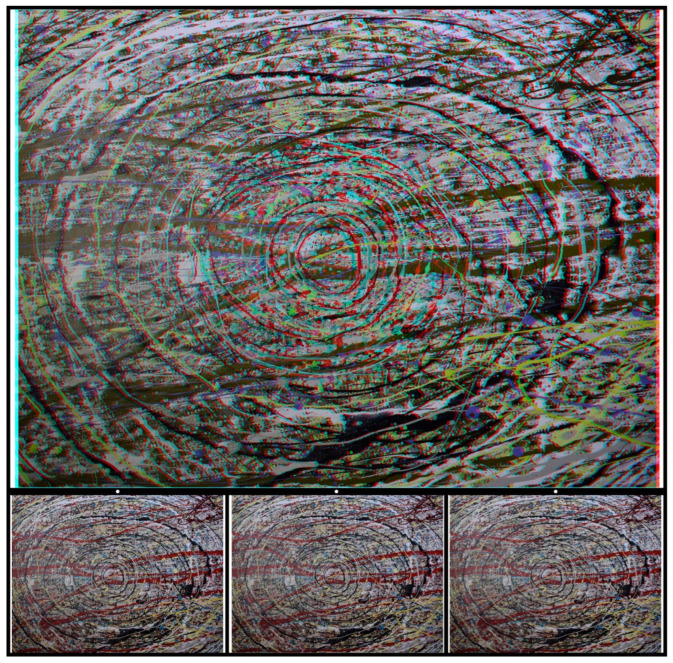
*Bull’s eye* by Nicholas Wade.

*Tachiste* paintings are well-suited to stereoscopic manipulations because of the dense contours within them. Figure 5 provides an example in which some structure is applied to the seemingly chaotic paint lines that stream across the surface: black lines surround a small central region, rather like a schematic eye. Two levels of stereoscopic depth are presented with the larger circle (like the iris) at a different plane to the small central circle (like the pupil). The dominant contours in the painting are the encircling black lines that give the impression of an eye. This is amplified by the central circular regions in stereoscopic depth.

## 3. Photographs

**Figure 6:**
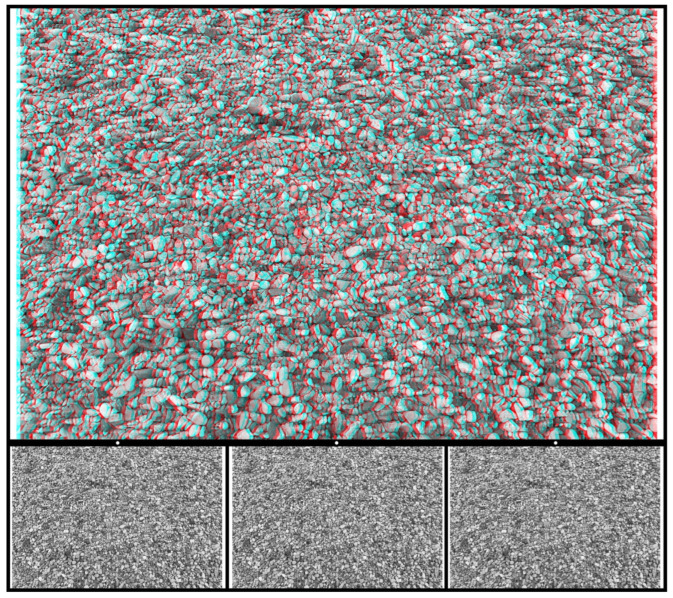
*DEPTH in stones* by Nicholas Wade.

All the carrier patterns above have been constructed with the help of a human hand, but many suitable alternatives can be found in the patterns that surround us. Photographs taken of textures like stones and plants can provide starting points for creating stereograms. Stone chippings are particularly good because they are approximately the same size but are irregularly dispersed (unlike RDSs) and have a high element density. Figure 6 is based on a photograph of small stones in which a word emerges in stereoscopic depth when viewed through red/cyan filters. The letters of the word DEPTH appear beyond the background with red/L and cyan/R, whereas they appear before it with cyan/L and red/R.

**Figure 7:**
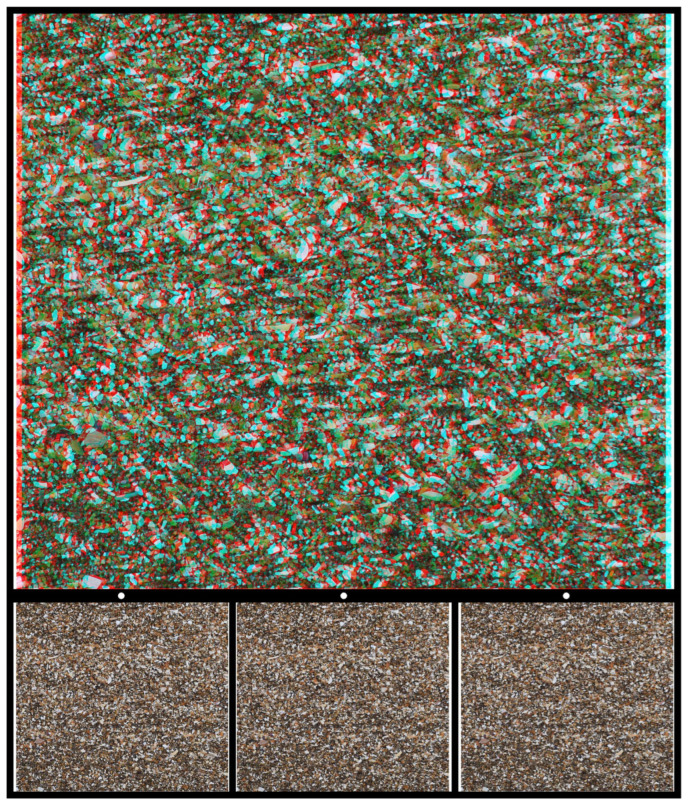
*Kanisza triangle in depth* by Nicholas Wade.

Similar characteristics can be found on beaches where a photograph of shell fragments formed the basis for Figure 7, which contains a familiar figure (used in countless books on vision) in stereoscopic depth. The three shapes seen in depth correspond to Kanisza’s figure in which the incomplete sides formed from the aligned sectors of circles appear to define the sides of a triangle. 

**Figure 8:**
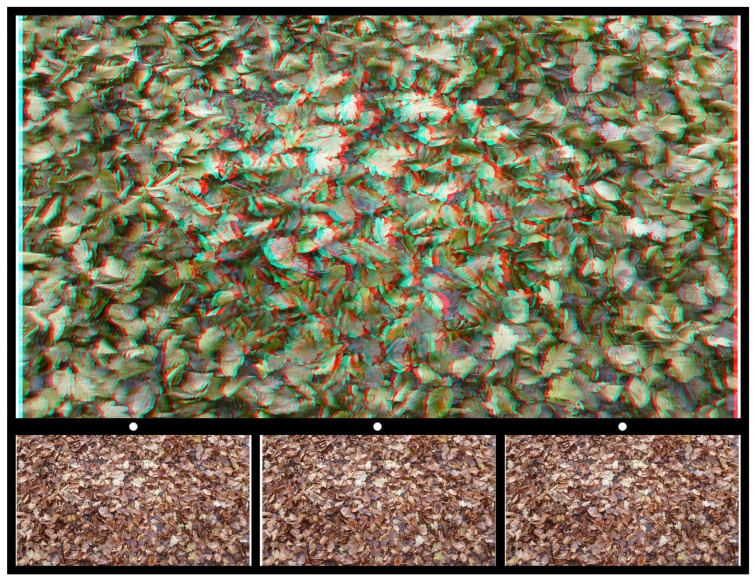
*Leaf fall* by Nicholas Wade.

Leaves, either growing on plants or having fallen to earth, provide patterns that can be employed in stereoscopic images. In Figure 8, a photograph of autumnal leaves provides the carrier pattern, and in the stereogram, the shape of a beech leaf is seen in depth lying either above the fallen leaves or beneath them.

## 4. Photographics

**Figure 9:**
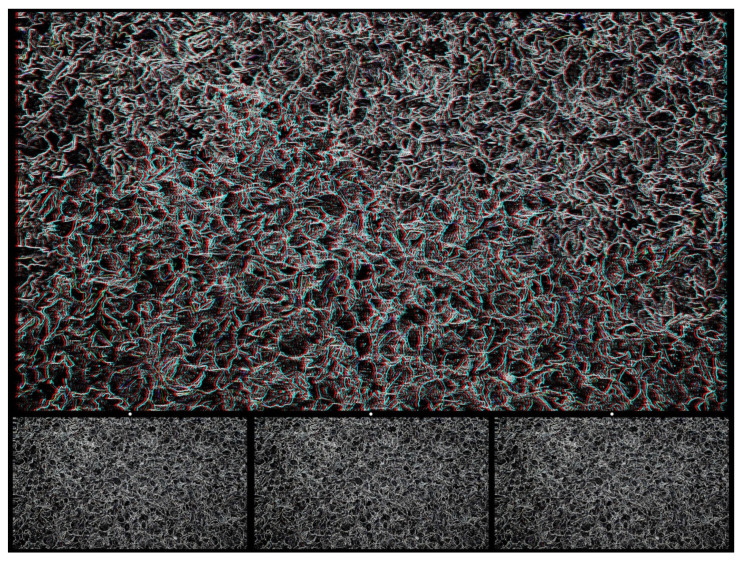
*Opera House II* by Nicholas Wade.

Photographics refers to the graphical manipulation of photographs that can lead to the creation of more complex and interesting carrier patterns for stereograms: their range can be greatly increased as will be evident in the following illustrations. In Figure 9, the starting points are two components shown in the figures above: the pattern of autumnal leaves (Figure 8) and the Sydney Opera House (Figure 3). The leaf pattern was converted to black-and-white and then rendered in line form leaving only the contours of the leaves; the silhouette of the Opera house was combined with the line pattern and laterally displaced; this was paired with an untransformed line pattern; and the negative of both were made into a stereogram.

**Figure 10:**
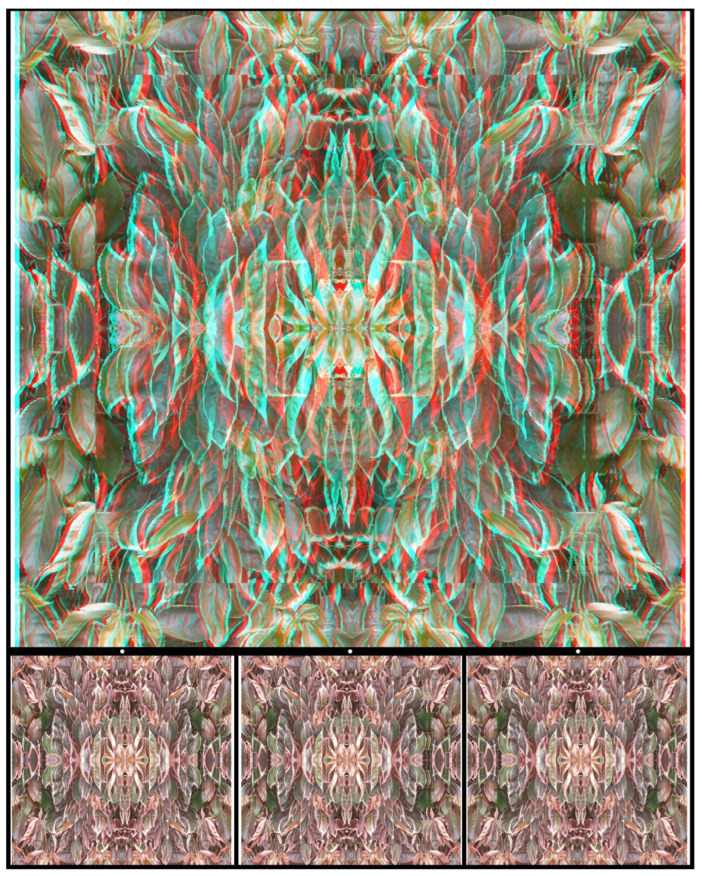
*Acalypha wilkesiana leaves* by Nicholas Wade.

Photographs can be multiplied to create patterns of symmetry that can be presented alone as carrier patterns or modified further. In Figure 10 the initial photograph of leaves has been repeated four times so that the overall pattern has vertical and horizontal axes of symmetry. The central pattern is emphasised by the stereoscopic circle in which it is seen and that, in turn, is within a larger square in a different depth plane.

**Figure 11:**
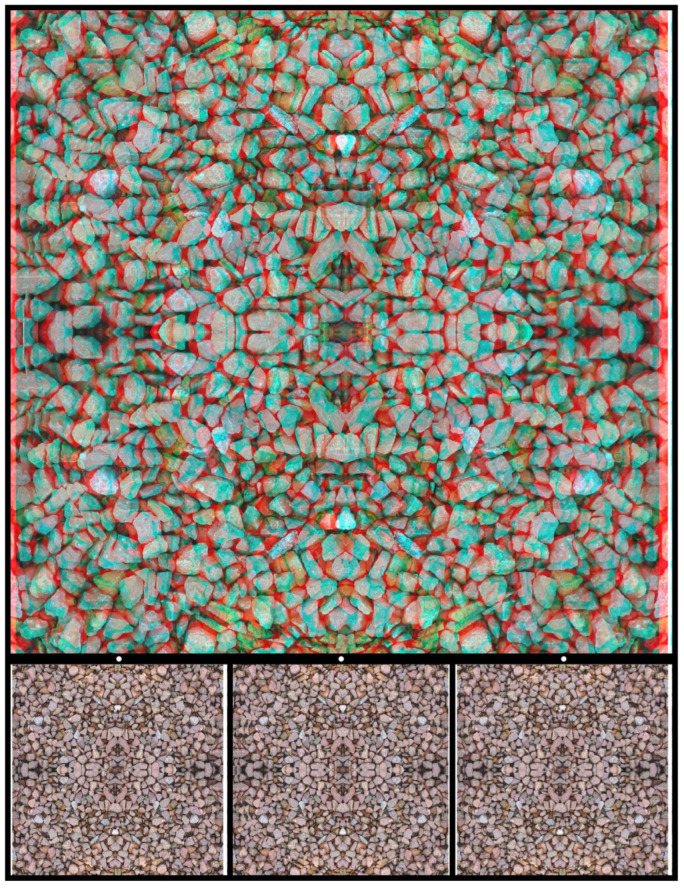
*Yin-Yang stones* by Nicholas Wade.

A similar procedure can be applied to photographs of stones. Those in Figure 11 have been combined via reflection and rotation and reveal, by binocular combination, a symbolic motif representing the union of opposites. In this case, the opposites are in different depth planes, both of which differ from that of the background.

**Figure 12:**
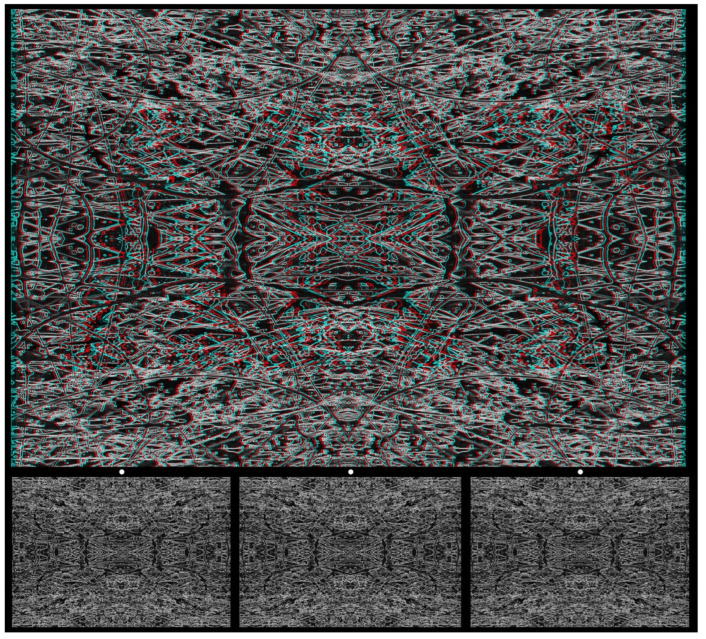
*Tachiste ART* by Nicholas Wade.

*Tachiste* paintings, like that in Figure 5, provide a fertile starting point for combining photography with graphics in creating a carrier pattern. Figure 12 is based on a photograph of detail from another painting, which was rendered in monochrome, and a line image was formed. It has a vertical and horizontal symmetry from the reflections and rotations of the image, and, finally, it was converted into a negative. The regions in the left image corresponding to letters of a word were displaced laterally relative to the background and paired with the untransformed right image. A word in stereoscopic depth emerges when viewed with red/cyan glasses or via the crossed or uncrossed combination of two of the lower images.

**Figure 13:**
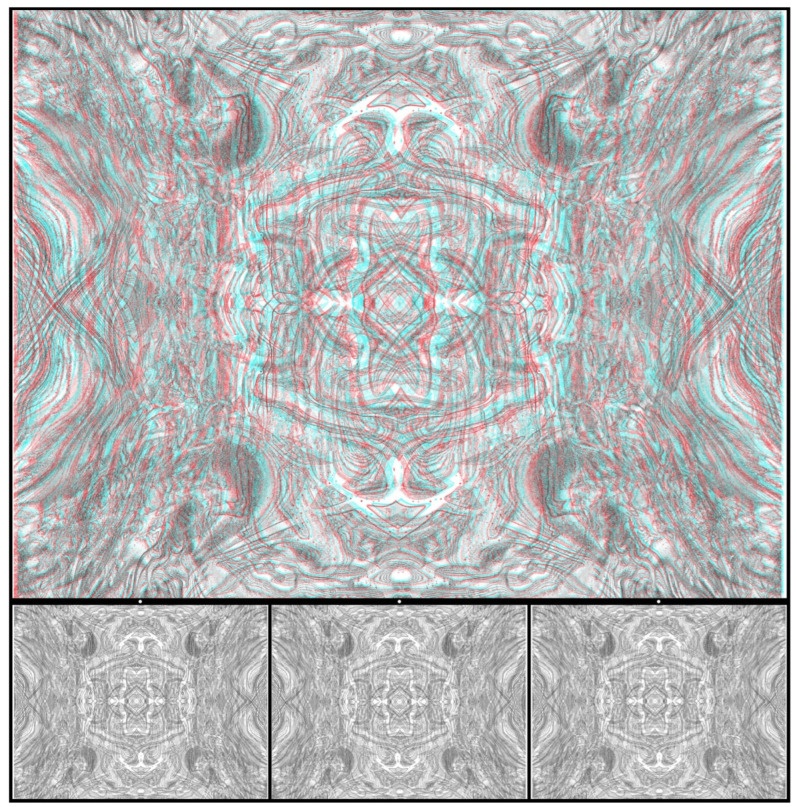
*Marbled hemicylinders* by Nicholas Wade.

In addition to *tachiste* paintings, those made with a technique similar to marbling can produce patterns with dense contours, suitable for modifications and making into carrier patterns. The processes involved in making the carrier pattern for Figure 13 are rather complex. Oil paints suspended on the surface of a pool of water flowed over a flat painted board, thereby allowing the different colours to intermingle. Details of the painting were photographed, digitized, and manipulated on the computer. The digital images were multiplied and combined in order to produce the symmetries that are evident in them. The image was rendered in black and white, and a line image was made and combined with the same pattern rotated by 90 deg. The horizontally extended feature in the centre of the design was emphasized by a stereoscopic ellipse with a horizontal major axis and the whole of one image was extended horizontally, resulting in the appearance of a curved surface that either approaches or recedes in depth.

**Figure 14:**
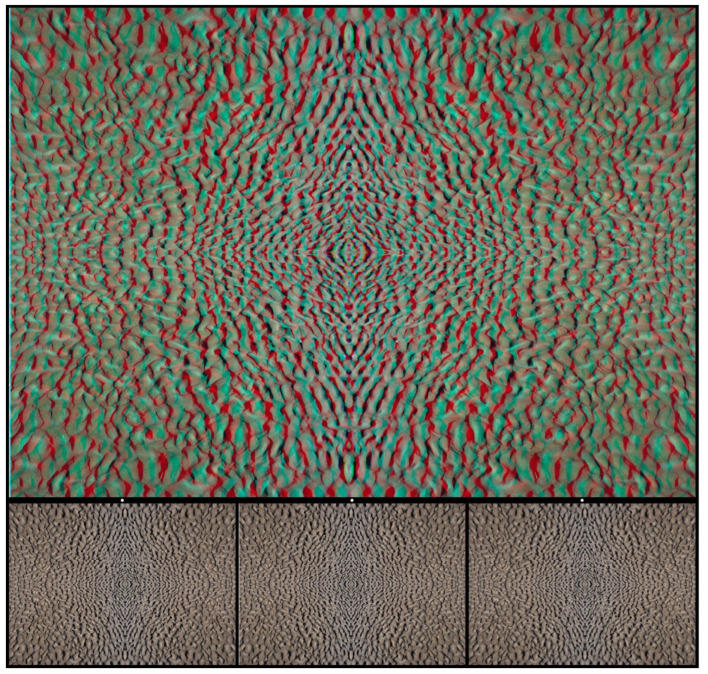
*Sun(glasses) and sand* by Nicholas Wade.

A photograph of ripples in the sand on a beach formed the basis for the stereogram in Figure 14. The pattern was multiplied to give vertical and horizontal axes of symmetry in which a pair of sunglasses can be seen in depth.

**Figure 15:**
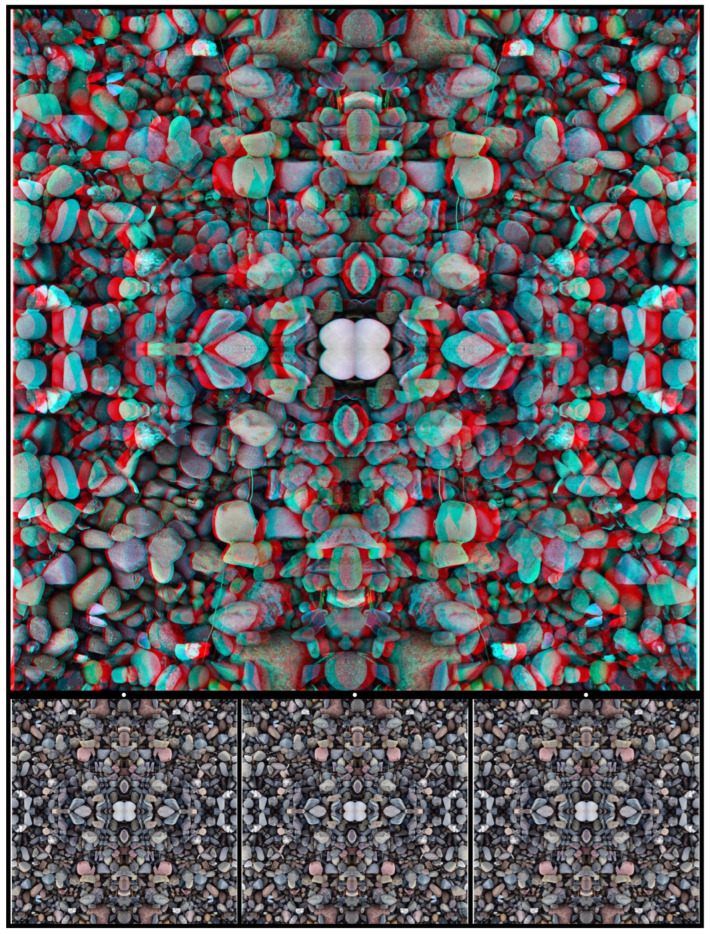
*Eternal staircase* by Nicholas Wade.

Multiple depth planes can be seen in Figure 15, which is based on a photograph of stones on a beach. It gives the impression of a spiral staircase with the individual steps radiating like petals from a central axis. Starting from a step at any level (when the depth emerges) and proceeding perceptually upwards or downwards, you return to the starting level. It is rather like an eternal staircase from which it is impossible to escape, hence the title. The ‘steps’ are formed from the overlapping regions of displaced semicircles with their diameters aligned with the four sides of the figure.

## 5. Conclusions

The study of stereoscopic depth perception was transformed by the invention and application of computer-generated RDSs by Julesz. Wheatstone’s dream of revealing to two eyes what was concealed from either one alone was realized. The impact of RDSs was such that alternative carrier patterns for disparities were rarely entertained let alone produced. Julesz witnessed and participated in the use of random-dot patterns in art, despite the simplicities and constraints of regular arrays of dots [16]. There are many ways of devising alternatives to random dots as carrier patterns. They consist of patterns with dense contours, usually with small irregular elements and without dominant features. Those shown in this article are manipulations of graphics, photographs, and their combinations, producing both carrier patterns for stereograms as well as having an attraction independent of their binocularity. Anaglyphs are particularly suited to such manipulations because of their colour components. Unlike mirror, lens, and prism stereoscopes, the combination of the two monocular images can be seen superimposed with anaglyphs when viewed without red/cyan glasses.

**Figure 16:**
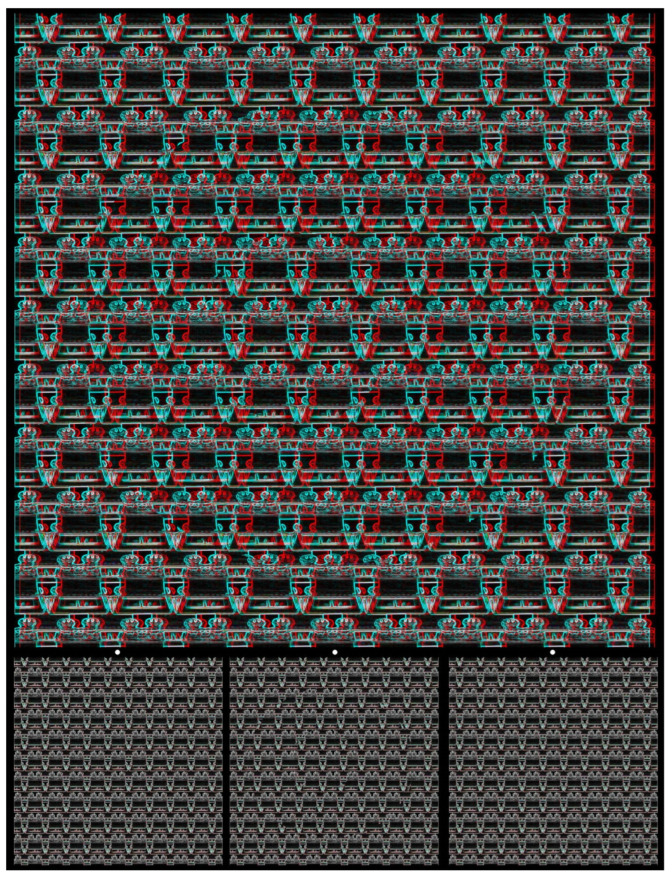
*Stereogram of stereoscopes* by Nicholas Wade.

Random-dot stereograms avoid recognition by either eye of the shapes in depth and even of the carrier pattern itself, the array of dots. As is evident from some of the illustrations above, recognition of the shape in depth does not preclude the recognition of the objects from which the carrier pattern was made. In fact, the elements of the carrier pattern can be intentionally recognizable as well as reflecting aspects of the concealed shapes. Perceptual puzzles can be included in the construction of the carrier patterns so that their constituent elements are not immediately evident. Both of these features can be seen in Figure 16. It is made up of a regular array of graphically manipulated lenticular stereoscopes and also carries shapes in depth that cannot be seen in the monocular images.

## Data Availability

No new data was created or analyzed in this study. Data sharing is not applicable to this article.
